# Tuning Magnetoelastic Transition and Magnetostrictive Properties of FeRh Alloy by Fe Substitution for Rh

**DOI:** 10.3390/ma19184012

**Published:** 2026-09-21

**Authors:** Minhan Yang, Xinhuai Wang, Wen Wang

**Affiliations:** School of Electronic Engineering, Xidian University, Xi’an 710071, China

**Keywords:** FeRh alloy, magnetoelastic transition, magnetic-field-induced metamagnetic transition, magnetostriction

## Abstract

FeRh alloys undergo an antiferromagnetic–ferromagnetic magnetoelastic transition accompanied by significant volume change, and thus exhibit giant magnetostriction. However, few studies have explored how Fe substitution for Rh affects the transition behavior, magnetostrictive properties, and the correlation between their respective variations in this system. Herein, we systematically explore the magnetoelastic transition behavior and magnetostrictive properties of the Fe_50+_*_x_*Rh_50−_*_x_* (*x* = 0, 0.25, 0.5) alloys. Experimental results demonstrate that the magnetoelastic transition temperature decreases, whereas both magnetostriction and its associated magnetic field response sensitivity increase with increasing Fe substitution. These findings provide an effective route to optimize the overall magnetostrictive performance of FeRh based alloys.

## 1. Introduction

Magnetostrictive materials can produce dimensional changes under an applied magnetic field [1,2,3,4]. They have attracted growing interest and been widely investigated for promising applications in sensors and actuators [5,6,7,8]. Among various magnetostrictive material systems, the alloys with magnetoelastic transition have attracted considerable attention due to the unique coupling between crystal volume and magnetic degrees of freedom [9,10,11]. In particular, FeRh alloys are considered a promising candidate due to their unique magnetoelastic transition and the associated giant magnetostriction [12,13,14].

FeRh undergoes a first-order metamagnetic transition from the antiferromagnetic (AFM) state to the ferromagnetic (FM) state upon heating, throughout which the crystal structure remains the ordered B2 (CsCl-type) symmetry but exhibits a pronounced unit-cell volume expansion of approximately 1% [15,16,17], a phenomenon known as the magnetoelastic transition [18,19]. The AFM-to-FM magnetoelastic transition can also be induced by magnetic fields and is accompanied by large magnetostriction [20,21,22]. Previous investigations have demonstrated that the transition behavior and magnetic ordering of FeRh alloys are strongly dependent on composition deviations [23,24,25]. However, limited studies have simultaneously explored how compositional variations influence both the transition behavior and magnetostrictive properties of FeRh alloys, as well as the correlation between their respective variations.

In this work, we systematically study the magnetoelastic transition and magnetostriction of the Fe_50+_*_x_*Rh_50−_*_x_* (*x* = 0, 0.25, 0.5) alloy system, where slight substitution of Rh by Fe is adopted. Our results reveal that the transition behavior and the magnetostrictive response are strongly modulated by Fe content. In particular, the magnetostriction and its magnetic field sensitivity are effectively enhanced by increasing the content of Fe. These findings may help guide the development of FeRh alloys with improved magnetostrictive performance.

## 2. Materials and Methods

Alloy ingots with nominal compositions of Fe_50+_*_x_*Rh_50−_*_x_* (0 ≤ *x* ≤ 0.5, compositions given in atomic percent (at.%)) were prepared by arc melting high-purity (99.9%) Fe and Rh (Beijing Zhongjinyan New Materials Technology Co., Ltd., Beijing, China) under an atmosphere of high-purity argon (Shaanxi Xilanfengtai Petrochemical Co., Ltd., Xi’an, China). The obtained ingots were spark-cut into appropriate dimensions, mechanically polished, and sealed in evacuated quartz tubes. Subsequently, they are annealed at 1273 K for 48 h, followed by quenching into room-temperature water to ensure compositional homogeneity and atomic ordering. The magnetoelastic transition temperature was determined by differential scanning calorimetry (DSC, Q2000, TA Instruments, New Castle, DE, USA) and dynamic mechanical analysis (DMA, Q800, TA Instruments). DSC measurements were performed using ~40 mg samples over 150–450 K at 10 K·min^−1^ under Ar. DMA measurements were conducted on strip-shaped specimens with dimensions of 15 × 1.5 × 1 mm^3^ in three-point bending mode at 0.2/0.4/1/4/10/20 Hz and 2 K·min^−1^ during cooling. The temperature dependence of normalized resistivity (Δρ/ρ-T, where Δρ/ρ = (ρ − ρ_0_)/ρ_0_, with ρ_0_ being the resistivity at 655 K) was measured over the temperature range of 125–655 K by a standard four-probe method in a Physical Property Measurement System (PPMS, Quantum Design, San Diego, CA, USA). The specimen dimensions for resistivity measurements are also 15 × 1.5 × 1 mm^3^, the applied measurement current was 0.5 mA, and the temperature sweep rate is 2 K·min^−1^. The temperature-dependent magnetization (M-T curve) and magnetic field-dependent magnetization (M-H curve) were characterized using a superconducting quantum interference device (SQUID, Quantum Design). XRD measurements were performed using a Bruker D8 Advance diffractometer (Bruker, Karlsruhe, Germany) with Cu K_α_ radiation, covering a 20–90° 2θ scan range with a step size of 0.02°. Magnetostriction (λ) measurements were carried out at various temperatures using strain gauge (KFLB, KYOWA, Tokyo, Japan) bonded to the sample surface with CC-33A strain gauge adhesive (KYOWA ELECTRONIC INSTRUMENTS Co., Ltd., Chofu, Japan).

Alloy ingots with nominal compositions of Fe_50+_*_x_*Rh_50−_*_x_* (0 ≤ *x* ≤ 0.5, compositions given in atomic percent (at.%)) were prepared by arc melting high-purity (99.9%) Fe and Rh (Beijing Zhongjinyan New Materials Technology Co., Ltd., Beijing, China) under an atmosphere of high-purity argon (Shaanxi Xilanfengtai Petrochemical Co., Ltd., Xi’an, China). The obtained ingots were spark-cut into appropriate dimensions, mechanically polished, and sealed in evacuated quartz tubes. Subsequently, they are annealed at 1273 K for 48 h, followed by quenching into room-temperature water to ensure compositional homogeneity and atomic ordering. The magnetoelastic transition temperature was determined by differential scanning calorimetry (DSC, Q2000, TA Instruments, New Castle, DE, USA) and dynamic mechanical analysis (DMA, Q800, TA Instruments). DSC measurements were performed using ~40 mg samples over 150–450 K at 10 K·min^−1^ under Ar. DMA measurements were conducted on strip-shaped specimens with dimensions of 15 × 1.5 × 1 mm^3^ in three-point bending mode at 0.2/0.4/1/4/10/20 Hz and 2 K·min^−1^ during cooling. The temperature dependence of normalized resistivity (Δρ/ρ-T, where Δρ/ρ = (ρ − ρ_0_)/ρ_0_, with ρ_0_ being the resistivity at 655 K) was measured over the temperature range of 125–655 K by a standard four-probe method in a Physical Property Measurement System (PPMS, Quantum Design, San Diego, CA, USA). The specimen dimensions for resistivity measurements are also 15 × 1.5 × 1 mm^3^, the applied measurement current was 0.5 mA, and the temperature sweep rate is 2 K·min^−1^. The temperature-dependent magnetization (M-T curve) and magnetic field-dependent magnetization (M-H curve) were characterized using a superconducting quantum interference device (SQUID, Quantum Design). XRD measurements were performed using a Bruker D8 Advance diffractometer (Bruker, Karlsruhe, Germany) with Cu K_α_ radiation, covering a 20–90° 2θ scan range with a step size of 0.02°. Magnetostriction (λ) measurements were carried out at various temperatures using strain gauge (KFLB, KYOWA, Tokyo, Japan) bonded to the sample surface with CC-33A strain gauge adhesive (KYOWA ELECTRONIC INSTRUMENTS Co., Ltd., Chofu, Japan).

## 3. Results and Discussion

**Figure 1:**
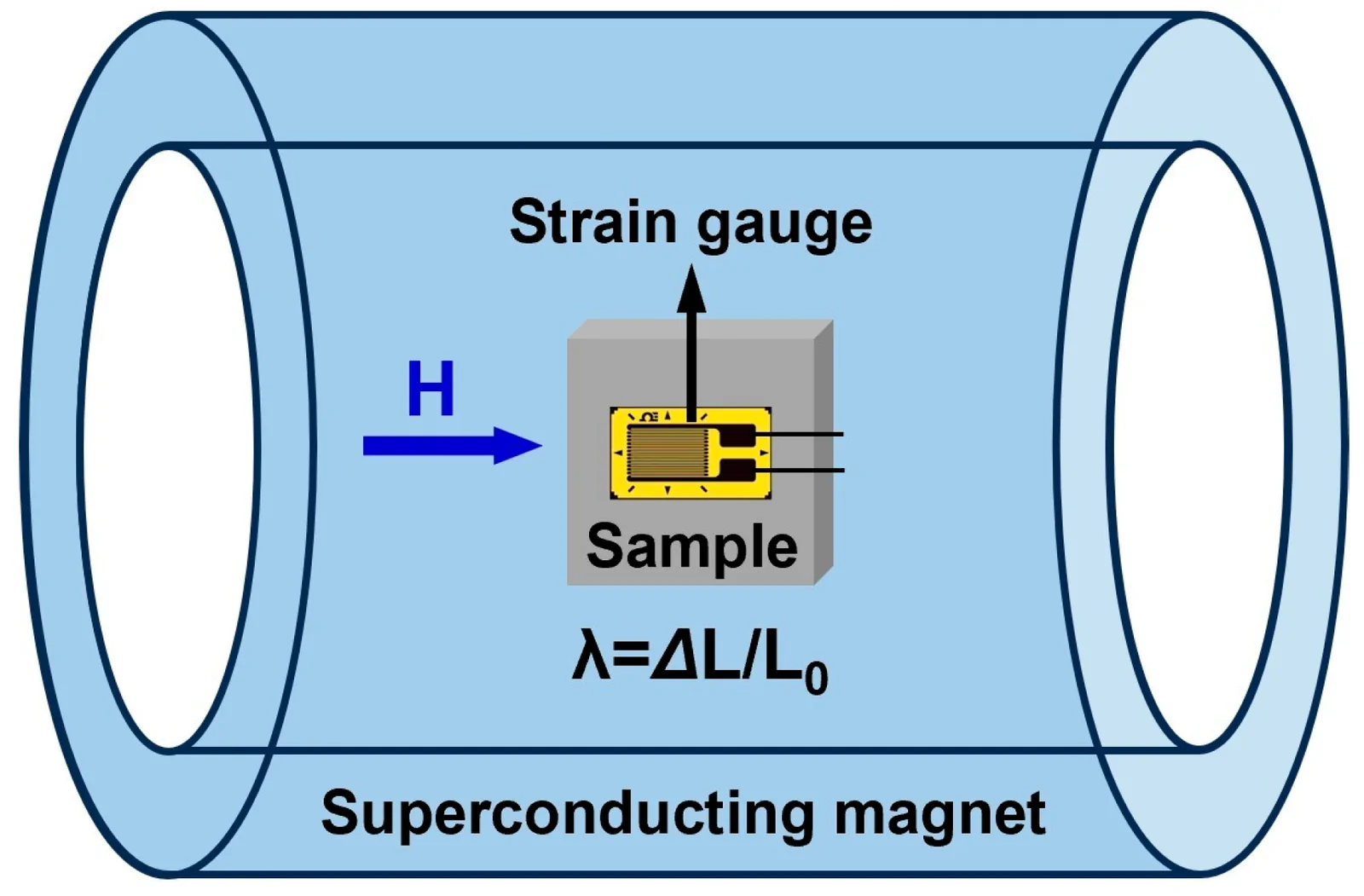
Schematic illustration for the magnetostriction measurement.

Figure 1 provides a schematic illustration for the magnetostriction measurement, where the strain gauge direction is aligned parallel to the applied magnetic field. The strain was defined as λ(H) = ΔL/L_0_ = (L(H) − L(0))/L(0), where L(0) and L(H) are the gauge lengths along the gauge direction at zero magnetic field and a given applied magnetic field H, respectively. The strain values were obtained from the resistance change in the strain gauge using the calibrated gauge factor provided by the manufacturer. Before each measurement, the sample was stabilized at the target temperature under zero field and equilibrated for 30 min. The magnetic field was swept from 0 to 20 kOe at 200 Oe·s^−1^ with three magnetization–demagnetization cycles at each temperature. The maximum magnetostriction sensitivity (dλ/dH)_max_ was obtained by the maximum slope of the measured λ-H data.

**Figure 2:**
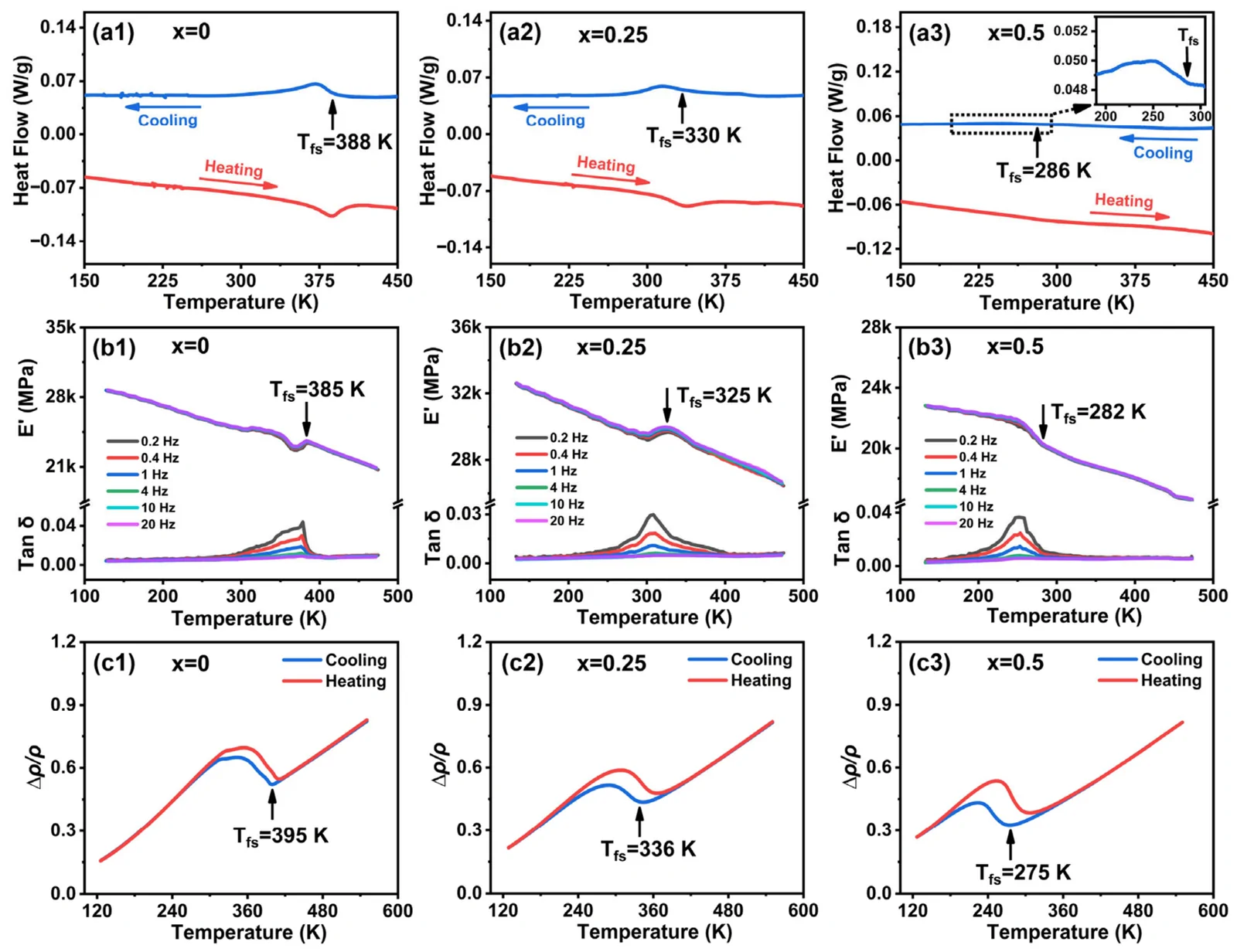
Temperature-dependent phase transition characteristics of Fe_50+_*_x_*Rh_50−_*_x_* alloys (*x* = 0, 0.25, 0.5). (**a1**–**a3**) Differential scanning calorimetry (DSC) curves measured during cooling and heating cycles. The inset in (**a3**) shows enlarged view of the transition region. (**b1**–**b3**) Dynamic mechanical analysis (DMA) results measured during cooling. (**c1**–**c3**) Temperature dependence of normalized resistivity (Δ*ρ*/*ρ*) measured during cooling and heating processes. The arrows indicate the magnetoelastic transition temperature (*T*_fs_).

Figure 2 presents the thermal, mechanical, and transport properties of Fe_50+_*_x_*Rh_50−_*_x_* alloys with *x* = 0, 0.25, and 0.5, revealing the evolution of the first-order magnetoelastic transition behavior with increasing Fe content. For a first-order transition, a two-phase coexistence temperature window exists. Accordingly, the forward transition upon cooling possesses a start temperature and a finish temperature. Meanwhile, first-order transitions exhibit thermal hysteresis, so the corresponding reverse transition upon heating also has its start and finish temperatures. These four characteristic transition temperatures can be determined from the inflection points of the DSC, DMA, and resistivity anomalies. In this work, we selected the start temperature *T*_fs_ of the forward transition upon cooling to characterize the transition temperature of FeRh alloys.

The DSC curves in Figure 2(a1–a3) demonstrate that all samples show obvious exothermic and endothermic peaks with relatively broad thermal hysteresis between cooling and heating processes, which is the typical feature of the first-order magnetoelastic transition in FeRh alloys [26,27]. During the cooling process, the high-temperature FM state with larger unit cell transforms into the low-temperature AFM state of smaller unit cell, which is accompanied by a decrease in entropy and gives rise to the exothermic peak. Upon heating, the reverse transition takes place and produces the endothermic peak. The thermal hysteresis originates from the nucleation energy barrier associated with the magnetoelastic transition. The corresponding transition temperature *T*_fs_ gradually decreases from 388 K for *x* = 0 to 330 K for *x* = 0.25, and further to 286 K for *x* = 0.5, revealing that increasing Fe content can effectively suppress the transition temperature.

The DMA properties shown in Figure 2(b1–b3) further verify the occurrence of the first-order magnetoelastic transition. A pronounced abnormal decline in the storage modulus (*E*′) appears near *T*_fs_, accompanied by a sharp peak in internal friction (tan *δ*). The storage modulus anomaly reflects lattice softening upon the magnetoelastic transition, while the sharp internal friction peak originates from energy dissipation induced by the motion of FM-AFM phase interfaces in the transition regime. Figure 2(c1–c3) display the temperature dependence of normalized resistivity (Δ*ρ*/*ρ*). All samples exhibit an obvious step in resistivity curve at *T*_fs_ upon cooling and heating processes, reflecting the fact that the magnetoelastic transition is associated electronic structure change [28,29,30]. The band structure reconstruction upon the transition of FeRh based alloys produces a substantial rise in carrier density when switching from the AFM to the FM state, which further accounts for the lower resistivity of the FM state relative to its AFM counterpart [29]. Similarly to the DSC results, the DMA and resistivity measurements also demonstrate that *T*_fs_ shifts toward lower temperature with increasing Fe concentration.

**Figure 3:**
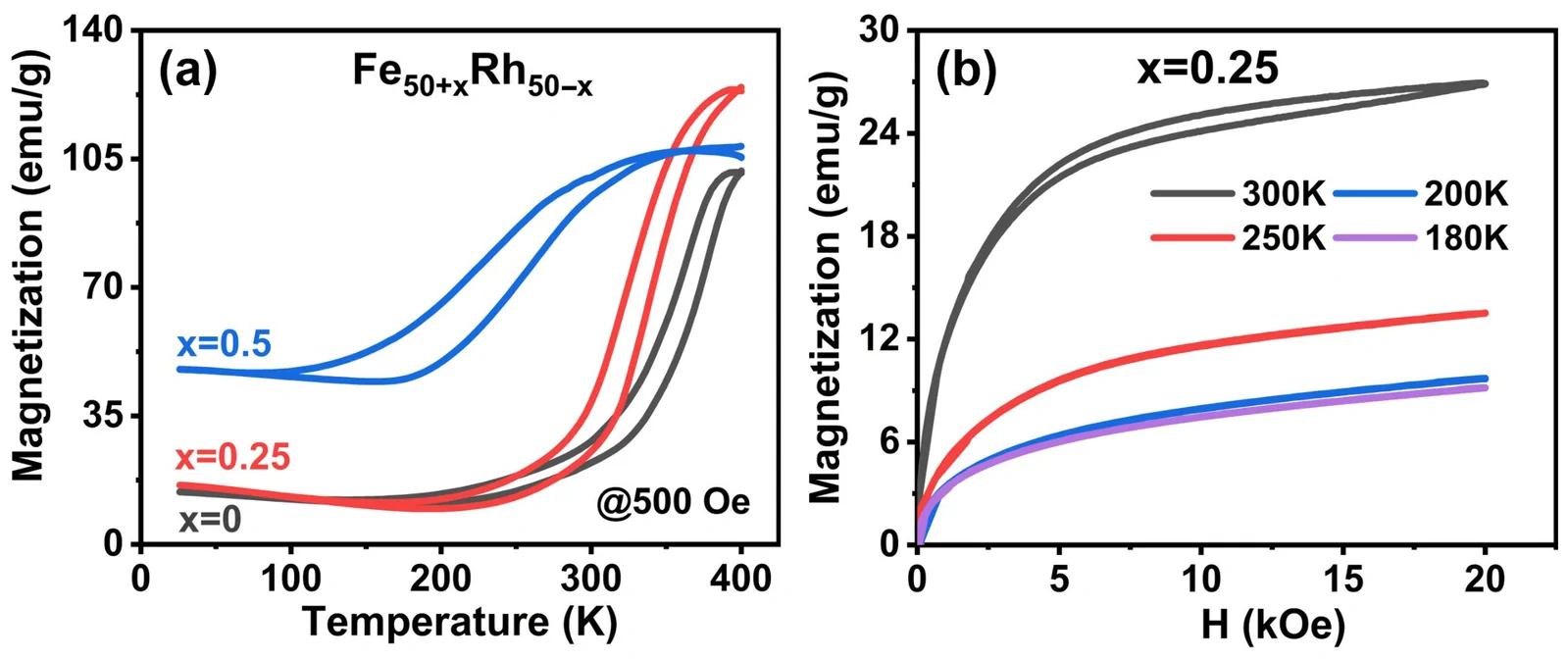
(**a**) Temperature dependence of magnetization measured under an applied magnetic field of 500 Oe during cooling and heating processes. (**b**) *M*-*H* curve of the Fe_50.25_Rh_49.75_ (*x* = 0.25) alloy.

To further elucidate the magnetic state evolution associated with the phase transition, temperature-dependent magnetization measurements were performed under an external magnetic field of 500 Oe, as presented in Figure 3a. All the FeRh samples are characterized by a significant decrease in magnetization during cooling, corresponding to the metamagnetic magnetoelastic transition from the ferromagnetic (FM) state to the antiferromagnetic (AFM) state in the system [31,32,33]. The change in magnetic state originates from the variation in interatomic distances: the short atomic distance in the compact unit cell at low temperature favors AFM coupling between Fe atoms, while the thermally expanded unit cell modifies the exchange integrals and strengthens the Fe-Rh interatomic FM interaction at high temperature [34,35]. Notably, a relatively broad transition temperature region can be clearly observed during the transition process, where the magnetization magnitude lies between those of the high-temperature FM state and the low-temperature AFM state, demonstrating the coexistence of FM and AFM states [36,37,38,39]. During the transition of FeRh alloys, domains of one magnetic phase nucleate initially and then tend to grow continuously. However, their subsequent growth is kinetically hindered by the surrounding untransformed regions, resulting in a broad two-phase coexistence region for the two magnetic states [40].

**Figure 4:**
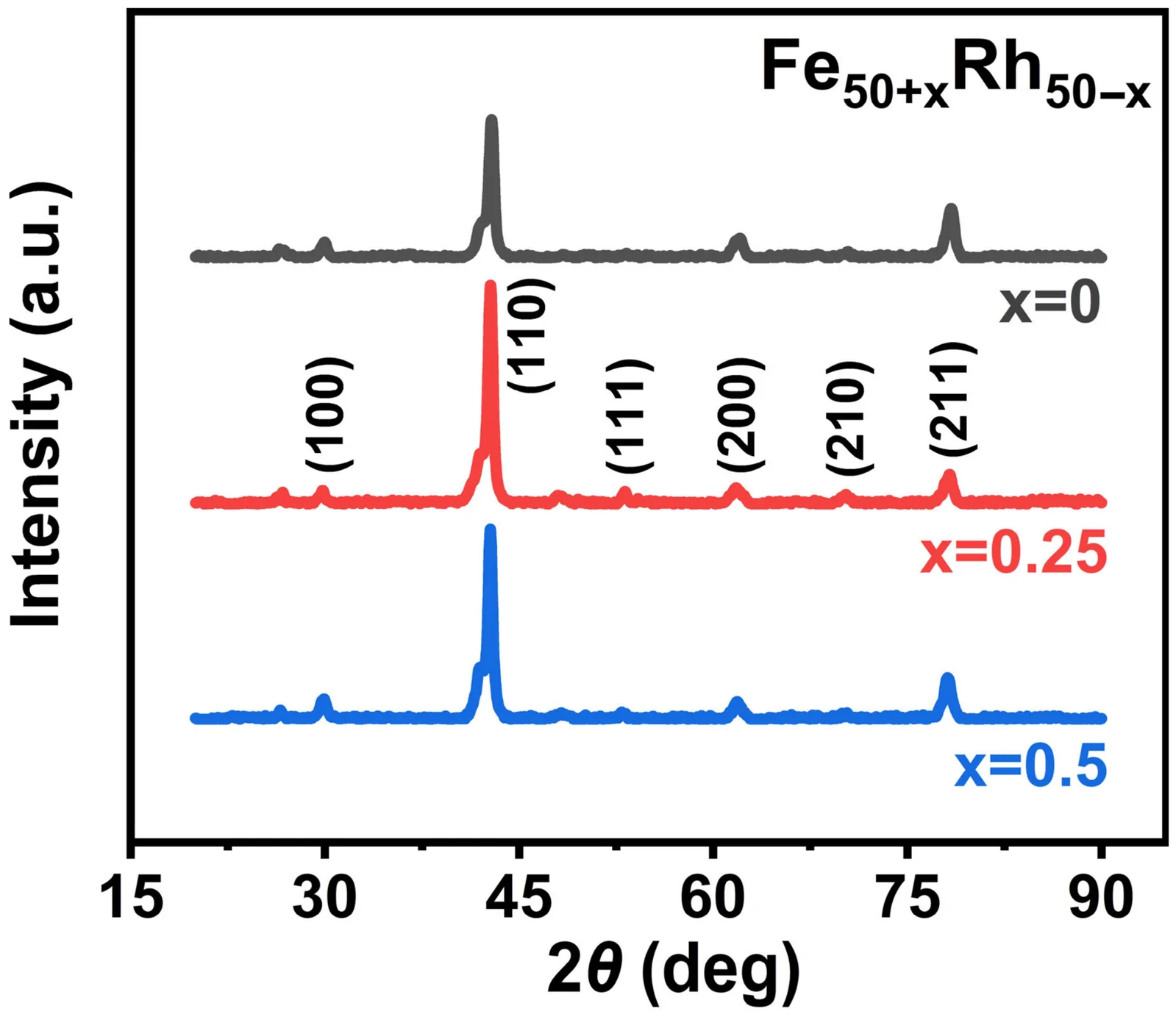
Room-temperature XRD patterns of Fe_50+_*_x_*Rh_50−_*_x_* alloys for *x* = 0, 0.25, and 0.5.

Figure 4 displays the X-ray diffraction (XRD) patterns of Fe_50+_*_x_*Rh_50−_*_x_* alloys measured at room temperature, which lies within or near the magnetoelastic transition region. The *x* = 0 and *x* = 0.25 samples are predominantly in the AFM state, whereas the *x* = 0.5 sample mainly remains in the FM state. Despite these samples staying in different magnetic ordering states, their diffraction patterns can be indexed to the same crystal symmetry, i.e., the B2 (CsCl-type) structure [41,42].

**Figure 5:**
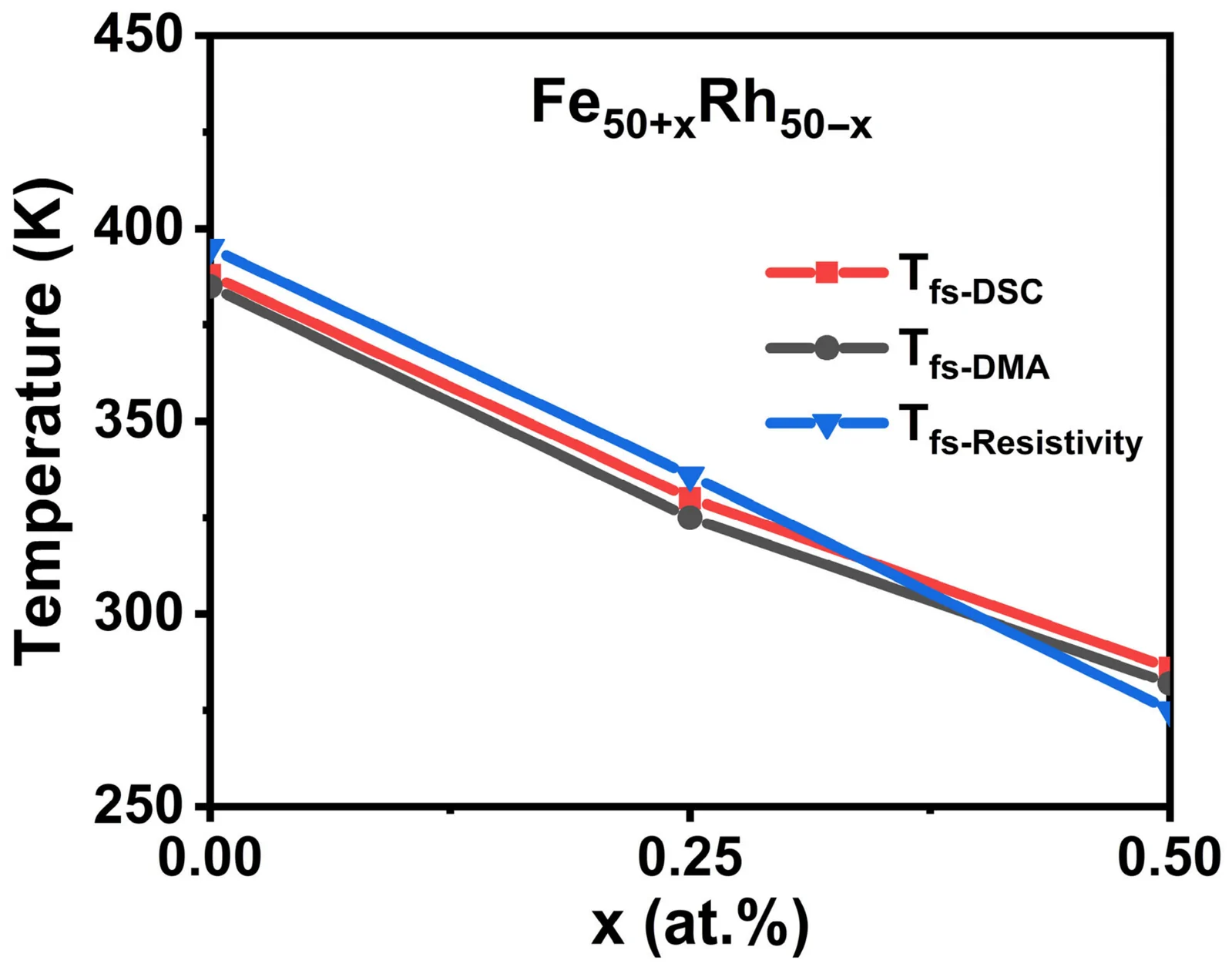
Start temperatures *T*_fs_ of the forward transition as a function of nominal Fe content for the Fe_50+_*_x_*Rh_50−_*_x_* (*x* = 0, 0.25, 0.5) alloy system, derived from DSC, DMA, and resistivity measurements upon cooling. The connecting lines are guides to the eye.

Based on the start temperatures of the forward transition determined from DSC, DMA, and resistivity measurements upon cooling, *T*_fs_ as a function of the nominal Fe content for the Fe_50+_*_x_*Rh_50−_*_x_* alloy system is summarized in Figure 5. It shows that the *T*_fs_ values obtained by the three above-mentioned measurement methods differ by several degrees. Such discrepancies are reasonable for different measurement techniques. To ensure consistency throughout our analyses, we used the *T*_fs_ values derived from DSC curves for all subsequent analyses. The transition temperature *T*_fs_ decreases with increasing Fe content. This observation is consistent with the results reported in previous investigations [24,25]. Mössbauer-spectroscopy investigations demonstrate that local hyperfine fields can be induced by Fe-antisite defects in FeRh alloys [43,44,45]. Moreover, previous work has established that the AFM interaction between Fe atoms is mediated by Rh atoms in the low-temperature AFM phase of FeRh alloys [46]. Fe substitution for Rh reduces the number of these Rh-mediated exchange paths and weakens the AFM coupling. Thus, the low-temperature AFM phase becomes unstable, leading to a decrease in the transition temperature.

**Figure 6:**
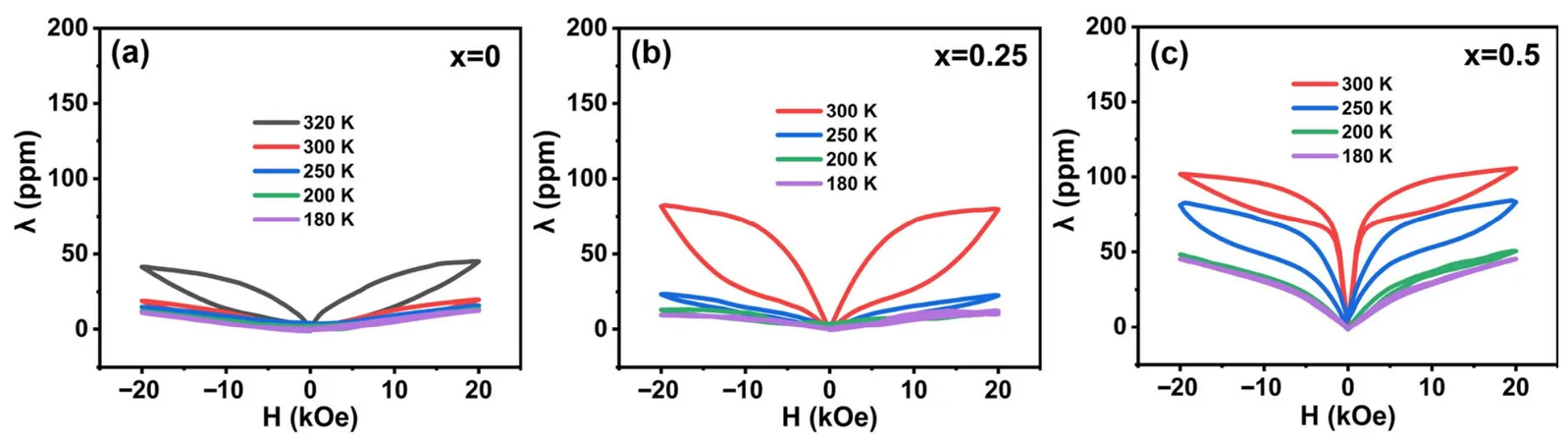
Magnetostriction (*λ*) vs. magnetic field (*H*) curves for Fe_50+_*_x_*Rh_50−_*_x_* alloys measured at different temperatures (≤*T*_fs_): (**a**) *x* = 0, (**b**) *x* = 0.25, (**c**) *x* =0.5.

Figure 6 shows the magnetostriction (*λ*) vs. magnetic field (*H*) curves of Fe_50+_*_x_*Rh_50−_*_x_* alloys measured at various temperatures (≤*T*_fs_). For the *x* = 0 sample (Figure 6a), a typical butterfly-shaped magnetostriction loop with large magnetic field hysteresis appears at 320 K. This temperature lies near its magnetoelastic transition temperature window, where the FM and AFM states exhibit comparable phase stability. In this situation, the magnetic field-induced transition from the AFM state to the FM state can easily take place, thereby resulting in a magnetic field-induced longitudinal strain together with pronounced field hysteresis. In contrast, at 300 K (<*T*_fs_) and lower temperatures (250, 200, and 180 K), the sample stays in a relatively stable AFM state, where the metamagnetic magnetoelastic transition is difficult to be induced by magnetic field. This gives rise to conventional magnetostriction featuring narrow V-shaped profiles, low magnetostriction magnitudes, and weak hysteresis. Similarly, the *x* = 0.25 alloy (Figure 6b) exhibits a butterfly-shaped magnetostriction curve at 300 K, a temperature close to its transition temperature window. Its corresponding *M*-*H* curve also displays a hysteresis loop (Figure 3b), further demonstrating that the magnetic field-induced transition can occur near its transition temperature window. As the temperature decreases to well below *T*_fs_ (250 K, 200 K, and 180 K), its magnetostriction curves transform into narrow V-shaped profiles. The corresponding *M*-*H* curves (Figure 3b) exhibit nearly no hysteresis, indicating that the metamagnetic magnetoelastic transition is barely triggered by the magnetic field well below *T*_fs_. Notably, the *x* = 0.5 alloy (Figure 6c) show a sharp rise in magnetostriction with increasing field, forming butterfly-shaped magnetostriction curves with small hysteresis at 300 K and 250 K, which lie near its transition temperature window. This rapid increase in magnetostriction is clearly different from the behavior observed in the *x* = 0 and *x* = 0.25 alloys. At 200 K (<*T*_fs_) and 180 K (<*T*_fs_), the sample stabilizes in the AFM state, and the magnetostriction curves revert to narrow V shapes.

**Figure 7:**
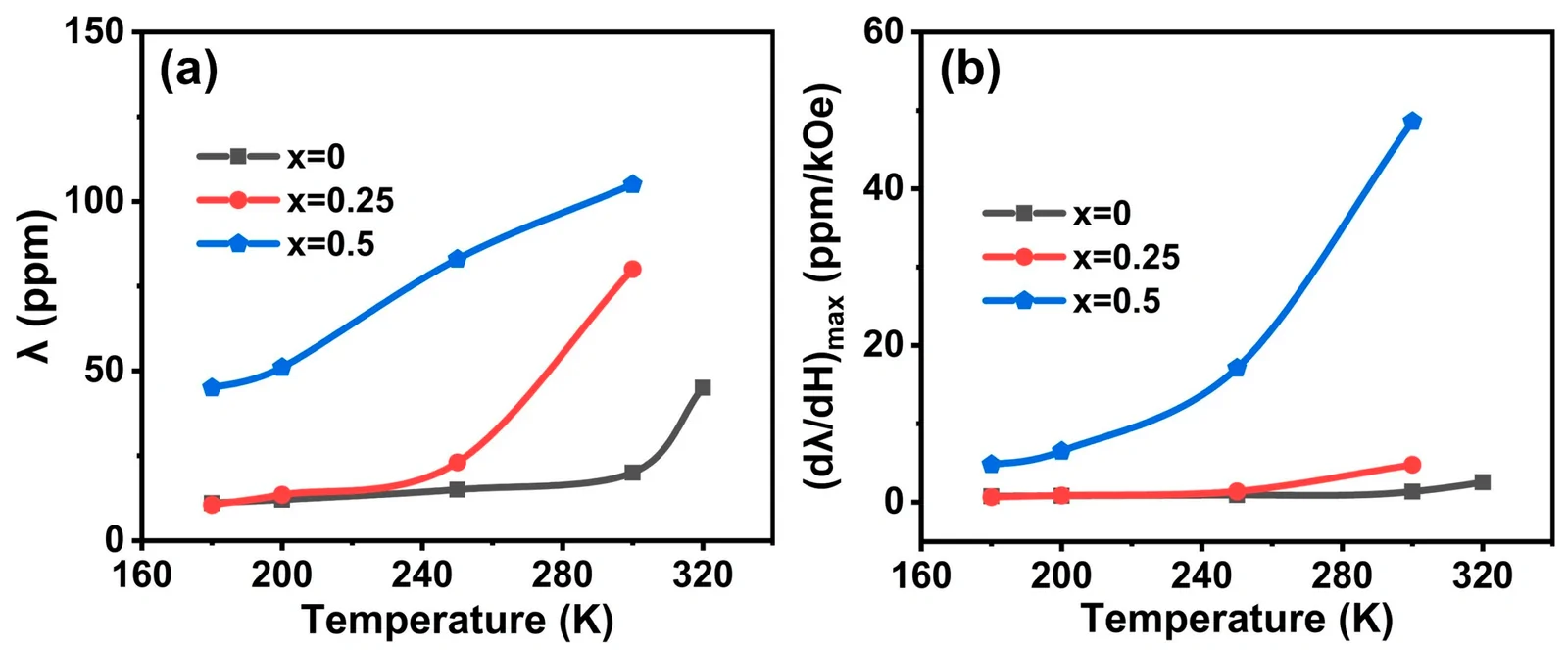
Temperature and composition dependence of the magnetostrictive properties of Fe_50+_*_x_*Rh_50−_*_x_* alloys. (**a**) Magnetostriction value (*λ*), (**b**) Maximum magnetostriction sensitivity (*dλ*/*dH*)_max_ as a function of temperature for samples with *x* = 0, 0.25, and 0.5.

Figure 7a,b systematically illustrate the temperature dependence of magnetostrictive properties including magnetostriction value (*λ*) and maximum magnetostriction sensitivity (*dλ*/*dH*)_max_ in Fe_50+_*_x_*Rh_50−_*_x_* alloy system. For all the samples (*x* = 0, 0.25 and 0.5), both the *λ* and (*dλ*/*dH*)_max_ gradually increase with increasing temperature when *T* ≤ *T*_fs_. This behavior results from the decreasing free-energy difference between the AFM and FM states as the temperature approaches *T*_fs_ [47], facilitating magnetic field-induced metamagnetic magnetoelastic transition. Consequently, both magnetostriction and field response sensitivity rise significantly with increasing temperature.

Figure 7a,b also show that both the *λ* and (*dλ*/*dH*)_max_ increase with increasing Fe content. This demonstrates that substitution of Fe for Rh significantly improves magnetostriction and its field response sensitivity. Such an effect can be attributed to the fact that Fe substitution for Rh introduces locally strengthened FM interactions and local lattice distortions, which facilitates the nucleation and growth processes of the magnetic field-induced metamagnetic magnetoelastic transition [24,47], resulting in a pronounced improvement in both *λ* and (*dλ*/*dH*)_max_. A maximum magnetostriction of 105 ppm is achieved for the Fe_50.5_Rh_49.5_ alloy, which is larger than that (60–80 ppm) of the classical FeCo magnetostrictive alloy [2]. These results demonstrate that the magnetostriction properties of FeRh alloy can be effectively improved by replacing costly Rh with inexpensive Fe, laying a foundation for further upgrading the overall performance of this alloy system.

## 4. Conclusions

In summary, the magnetoelastic transition behavior and magnetostrictive properties were systematically investigated in the Fe_50+_*_x_*Rh_50−_*_x_* (*x* = 0, 0.25, 0.5) alloy system. All alloys exhibit a typical metamagnetic magnetoelastic transition, and the transition temperature (*T*_fs_) decreases with increasing Fe content. The magnetostriction and its magnetic field response sensitivity increase as temperature rises from below *T*_fs_ up to near *T*_fs_, and both parameters improve with increasing Fe content. Accordingly, identical strain output can be achieved in these alloys under a lower applied magnetic field, which is beneficial for developing miniaturized magnetic devices. These findings reveal that compositional modification provides an effective strategy to tailor the magnetoelastic transition and magnetostrictive performance of FeRh alloys, offering potential for designing high-performance magnetostrictive materials.

## Data Availability

The original contributions presented in this study are included in the article. Further inquiries can be directed to the corresponding author.
